# Supplementary material for: An efficient blockchain-based authentication scheme with transferability
Source: PLoS One. 2024 Sep 12;19(9):e0310094. doi: 10.1371/journal.pone.0310094 (PMC11392242; doi:10.1371/journal.pone.0310094)
Supplement: Attachment — Submitted filename: Response to Reviewers.docx [file pone.0310094.s002.docx]

RESPONSE TO EDITOR AND REVIEWERS

**RESPONSE TO THE EDITOR:**

**EDITOR COMMENT**: Thank you for submitting your manuscript to PLOS ONE. After careful consideration, we feel that it has merit but does not fully meet PLOS ONE’s publication criteria as it currently stands. Therefore, we invite you to submit a revised version of the manuscript that addresses the points raised during the review process.

**RESPONSE**: Thank you for reviewing and providing feedback on our manuscript. We are very pleased to learn that our manuscript has some value. In response to the comments made during the review process, we have carefully revised and improved the manuscript. We are now submitting the revised manuscript, which we hope will meet the publication criteria of PLOS ONE.

======================================================================

**General Comments:**

**COMMENT**:

Please ensure that your manuscript meets PLOS ONE's style requirements, including those for file naming.

**RESPONSE**:

We have checked and ensured that the manuscript complies with all relevant formatting specifications and file naming conventions.
======================================================================

**COMMENT**:

Please note that PLOS ONE has specific guidelines on code sharing for submissions in which author-generated code underpins the findings in the manuscript. In these cases, all author-generated code must be made available without restrictions upon publication of the work.

**RESPONSE**:

Thank you for reminding us of PLOS ONE's specific guidelines on code sharing. We have added a description to the Data Availability Statement to ensure that the code is all unconditionally publicly accessible via GitHub.

======================================================================

**COMMENT**:

We noticed you have some minor occurrence of overlapping text with the following previous publication(s), which needs to be addressed: Securing Fog Computing with a Decentralised User Authentication Approach Based on Blockchain - https://doi.org/10.3390/s22103956 (among others) In your revision ensure you cite all your sources (including your own works), and quote or rephrase any duplicated text outside the methods section. Further consideration is dependent on these concerns being addressed.

**RESPONSE**:

Thank you for pointing out the overlap in our manuscript with the previously published article "Securing Fog Computing with a Decentralised User Authentication Approach Based on Blockchain." The overlapping part is due to a similar summary of the same referenced article, "CAB-IoT: Continuous authentication architecture based on Blockchain for Internet of Things," which is a coincidence. We have rewritten this section and cited all relevant sources, including our own works, in the revised manuscript.

**The overlapping parts are as follows:**

In our article: "Al-Naji et al. [29] proposed CAB-IoT, a distributed and scalable continuous authentication solution based on Blockchain technology."

In the mentioned article: "The authors in [30] proposed CAB-IoT, which is a distributed and scalable continuous authentication solution based on blockchain technology."

**The rewritten sentence is as follows:**

Al-Naji et al [29] proposed a distributed and scalable continuous authentication scheme implemented using blockchain technology.

**COMMENT**:

We note that your Data Availability Statement is currently as follows: "All relevant data are within the manuscript and its Supporting Information files." Please confirm at this time whether or not your submission contains all raw data required to replicate the results of your study. Authors must share the “minimal data set” for their submission. PLOS defines the minimal data set to consist of the data required to replicate all study findings reported in the article, as well as related metadata and methods (<https://journals.plos.org/plosone/s/data-availability#loc-minimal-data-set-definition>).

**RESPONSE**:

We have added a minimal dataset in the Supporting Information, which includes the data values underlying the metrics in our tables, as well as the data used for generating the figures. Furthermore, due to concerns about insufficient data, we computed and added additional data, and recalculated some values presented in the paper.

**COMMENT**:

Please include a copy of Table 6 which you refer to in your text on page 11.

**RESPONSE**:

We apologize for the confusion. This seems to be a misunderstanding. We have removed the reference to Table 6 in the revision. Therefore, we have revised the text accordingly in 6.3.

**RESPONSE TO THE REVIEWER1:**

**COMMENT**: The manuscript's structure was not well-prepared.

**RESPONSE**: Thank you for your feedback. We have addressed the issues you mentioned by making adjustments and improvements. We have added the missing key elements, shared datasets and code, and removed incorrect references. We have also clarified and organized unclear figures. We hope these improvements enhance the quality and comprehensibility of the manuscript.

======================================================================

**COMMENT**: The contribution in this manuscript is simple and has little novelty.

**RESPONSE**: Considering the limited research relying on smart contracts to achieve delegation of authentication permissions, we believe our study is innovative. Compared to similar studies (e.g., the approach proposed by Krenn et al. [18]), we have successfully reduced the gas consumption for a single authentication to less than one-third of the original scheme, achieving significant cost savings. In future research, we will also consider appropriate applications and relevant use cases for this approach.

======================================================================

**COMMENT**: Although the authors focused on the gas computation factor in their proposed system, they have not compared their system with those mentioned in the Related Works section.

**RESPONSE**: In Table 3, we list the deployment and verification costs of several blockchain-based authentication schemes. In addition to our ECDSA scheme, we compare it with four related works (SJ23 [31]; KL21 [18]; KN20 [30]; ZWY19 [22]) and the scheme proposed in this paper. To clearly articulate our contributions, we extensively revised section 5.4.

======================================================================

**COMMENT**: Some figures in the manuscript are not clear and the titles of their axis need to be clarified in the figures themselves.

**RESPONSE**: We have already made revisions to the manuscript. The figures are now clear, and the axis titles have been clearly labeled in the figures.

======================================================================

**COMMENT**: Some sections in the manuscripts don't contain any paragraphs such as (4.3 Notation).

**RESPONSE**: We have taken note and made corrections to ensure that all sections contain the necessary paragraphs and content, including section 4.3.

======================================================================

**COMMENT**: The study relied on three assumptions, some of which, like the third assumption, cannot be achieved in the real world.

**RESPONSE**: Considering the complexity of the real world, we recognize that assuming all communications are completely secure may be overly idealistic. Therefore, we modify the assumption: communications between users and agents employ appropriate security measures, such as using protocols like TLS, to ensure confidentiality and integrity of communications

======================================================================

**COMMENT**: The results obtained should be presented in a more comprehensible and lucid manner.

**RESPONSE**: In Section 5.4, we revised the description of the gas consumption analysis to make the results more comprehensible. In the conclusion, by comparing with data from existing studies, we highlighted the efficiency advantages of the proposed scheme in this research.

======================================================================

**RESPONSE TO THE REVIEWER2:**

**COMMENT**: Authors are asked to incorporate or explain the same with any one of the suitable application. Also list few suitable applications where this scheme can be incorporated.

**RESPONSE**: Thank you for your valuable feedback. We will provide a detailed explanation of the applicability of our scheme in practical applications and list some suitable application examples. In blockchain-based supply chain management, gas consumption is a critical factor due to the frequent execution of smart contracts. Our scheme optimizes gas consumption, significantly improving system efficiency. Reducing the gas required for transactions not only lowers operational costs but also enhances the scalability of the supply chain network, ensuring that the supply chain process remains cost-effective while maintaining transparency and security. In addition to supply chain management, our scheme has broad applications in decentralized finance (DeFi) platforms, healthcare data management, voting systems, digital identity verification, and intellectual property management.

======================================================================

**COMMENT**: This work mainly focus on hashing concept and computational complexity how this scheme will be helpful or suitable while scaling up the experiment in terms of no. of users ? Justify.

**RESPONSE**: Our design involves assigning a smart contract to each authorized user, even though users may have multiple different agents. The burden of a single smart contract depends on the number of authorized agents endorsing the authentication permissions, as delegation is one-time. For delegating authentication permissions to multiple users, we have considered consolidating them into a single smart contract. While this approach significantly increases gas consumption for deploying and invoking the smart contract, it can reduce the gas costs allocated to each user. However, ensuring fairness among users with varying numbers of agents and different frequencies of contract invocation is also a challenge that requires careful consideration.
